# Improving access to health care in a rural regional hospital in South Africa: Why do patients miss their appointments?

**DOI:** 10.4102/phcfm.v9i1.1255

**Published:** 2017-03-30

**Authors:** Lucy Frost, Louis S. Jenkins, Benjamin Emmink

**Affiliations:** 1 Thames Valley and Wessex Leadership Academy, United Kingdom; 2 Department of Anthropology, Durham University, United Kingdom; 3 George Regional Hospital, Western Cape Government, South Africa; 4 Division of Family Medicine and Primary Care, Stellenbosch University, South Africa; 5 George Regional Hospital, Western Cape Government, South Africa

## Abstract

**Background:**

Access to health services is one of the Batho Pele (‘people first’) values and principles of the South African government since 1997. This necessitated some changes around public service systems, procedures, attitudes and behaviour. The challenges of providing health care to rural geographically spread populations include variations in socio-economic status, transport opportunities, access to appointment information and patient perceptions of costs and benefits of seeking health care. George hospital, situated in a rural area, serves 5000 outpatient visits monthly, with non-attendance rates of up to 40%.

**Objectives:**

The aim of this research was to gain a greater understanding of the reasons behind non-attendance of outpatient department clinics to allow locally driven, targeted interventions.

**Methods:**

This was a descriptive study. We attempted to phone all patients who missed appointments over a 1-month period (*n* = 574). Only 20% were contactable with one person declining consent. Twenty-nine percent had no telephone number on hospital systems, 7% had incorrect numbers, 2% had died and 42% did not respond to three attempts.

**Results:**

The main reasons for non-attendance included unaware of appointment date (16%), out of area (11%), confusion over date (11%), sick or admitted to hospital (10%), family member sick or died (7%), appointment should have been cancelled by clerical staff (6%) and transport (6%). Only 9% chose to miss their appointment. The other 24% had various reasons.

**Conclusions:**

Improved patient awareness of appointments, adjustments in referral systems and enabling appointment cancellation if indicated would directly improve over two-thirds of reasons for non-attendance. Understanding the underlying causes will help appointment planning, reduce wasted costs and have a significant impact on patient care.

## Background

Access to health care is recognised as a fundamental human right globally.^1^ In 1997, the South African national government embarked on a Batho Pele campaign aimed at improving service delivery to the public. Batho Pele is a Sesotho phrase meaning ‘people first’, committing the public service to serve all the people of South Africa. Access to health care is one of the Batho Pele values and principles, and it is also enshrined as a basic human right in the National Health Insurance (NHI) White Paper (2015) of South Africa.^2^ For this approach to succeed, some changes need to take place around public service systems, procedures, attitudes and behaviour.

Healthcare 2030 has set out a vision statement for health care in the Western Cape Province, endorsing ‘Access to person-centred, quality care’.^3^ Access to health care has been widely debated, but one useful definition is ‘providing the right service at the right time in the right place’.^4^

George Regional Hospital (GRH) serves approximately 605 380 people from Eden and 73 336 people from Central Karoo, covering an area of around 62 185km.^5^ This poses challenges of how to provide access to care for such a geographically spread population. Confounding this further are issues of variation in socio-economic status, transport opportunities, access to appointment information and patient perceptions of costs and benefits of seeking health care.^6^

In July 2015, there were over 5000 outpatient visits to GRH. With such large numbers, when aiming to provide ‘access to person-centred, quality care’, the outpatient department (OPD) needs to be a focus. The frequency of ‘did not attend’ (DNA) rates for clinic appointments is an important measure of accessibility.^6^ Analysis of clinic attendance at GRH showed a variable DNA rate of between 20% and 40%, depending on clinic specialty, between July and October 2015. This high non-attendance rate makes it difficult to develop reliable safety nets, and many of these patients are never seen in OPD.

Despite the complexities of providing outpatient care, there are few studies on reasons for non-attendance at outpatient clinics in South Africa. Some of the reasons for DNAs in South African OPDs include lack of finances, migration, forgetting and long distances from home to clinic.^7,8,9^

Internationally, other factors have also been identified, including patient apathy, concern over investigation or seeing junior staff.^10,11^ Illness beliefs have also been shown to have a large impact on a patients’ choice to attend appointments.^12^

Although some of these challenges may be inherent in providing health care in a resource-limited setting, some interventions, such as reminder through short messaging systems (SMS), can be effective in reducing the rate of DNAs.^13^ An in-depth analysis of the major barriers could potentially allow targeted intervention leading to financial and administrative improvements,^14^ as well as ethical and clinical benefits of easier access to health care.^15^

The aim of this study was to explore factors contributing to patients missing their OPD appointments, and patients’ ideas of how to improve the accessibility of OPD, allowing the development of a more patient-centred health service.

## Methods

**FIGURE 1:**
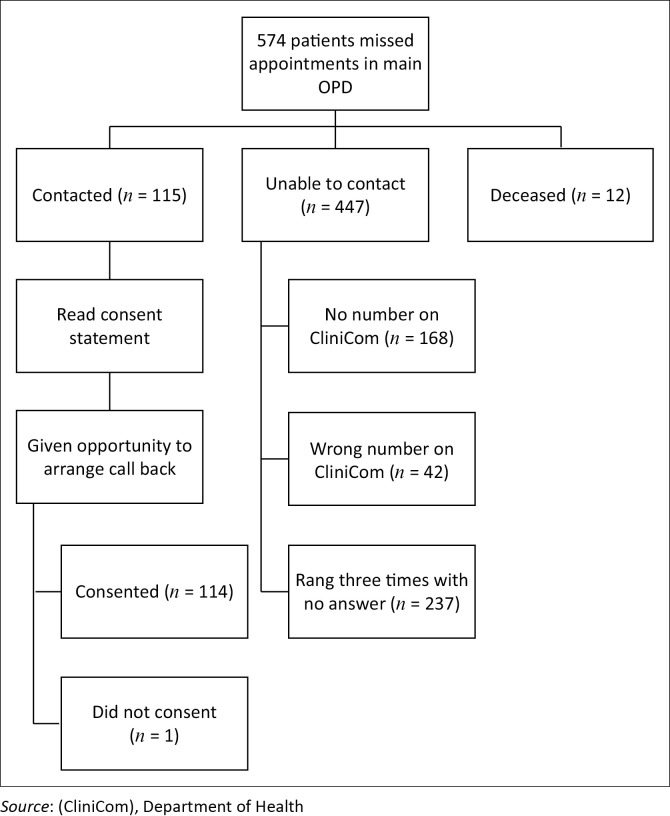
Outcomes of attempted patient contacts.

This was a descriptive study. A total of 574 patients did not attend their OPD appointment. Attempts were made to contact all patients, but only 115 were contacted. Ethics approval was obtained via Stellenbosch University Research Ethics Committee (N15/12/127). Semi-structured interviews were conducted telephonically with all adult patients who had missed appointments in the GRH OPD over a 1-month period in 2015, who were contactable and gave voluntary informed consent. Patients from specialties with OPD services in other areas of the hospital, and from obstetrics, were excluded. Telephonic interviews were chosen because of previously reported high response rates from this method^11^ and difficulties following patients up via other means. Patient details and telephone numbers were obtained through the hospital information system (CliniCom) (see Figure 1).

The interviews were semi-structured, using a combination of qualitative and quantitative questions, and were conducted by the first author, a clinician with health research experience. The interview guide contained questions about demographics, factors influencing non-attendance, perceptions of their appointment and their health and suggestions for improving the accessibility of OPD (available on request from the corresponding author). Interpreters were used where necessary to allow interviews to be conducted in the participants’ preferred language (English, Afrikaans or Xhosa). Several participants declined to answer specific questions, affecting the total numbers of responses for that question. For this reason, raw numbers are given so this is clear.

Analysis of quantitative interview data was done using descriptive statistics. Qualitative data were manually analysed using a thematic approach. Two researchers coded all qualitative responses. Codes were then compared and adjusted until there was an agreement among the researchers.

Anonymity was maintained through allocating each participant an ‘interview code’, which was the only identifier attached to the data. Interview data and participant details were stored separately, each in a password-protected file. Confidentiality was maintained, with no health care professionals aware of which patients participated in the study. In the case that someone other than the patient answered the phone, no patient details, clinical information or information about the purpose of the call was given. A scripted information and consent statement was read to each participant; opportunities were given for any questions, and patients were informed that they could withdraw consent at any point. All participants were given an opportunity to arrange a call at a later time to give them time to consider their involvement. Verbal consent was obtained before continuing with the interview.

## Results

There were 115 successful contacts from a total of 574 attempts, and 114 of these patients consented to participate (see Table 1). The mean age was 53.2 years (range 20–86).

**TABLE 1:** Characteristics of study participants.

Characteristics	Number of participants	Percentage of total participants (%)
Preferred language (*n* = 114)		
English	19	16.7
Afrikaans	72	63.2
Xhosa	23	20.2
Gender (*n* = 113)†		
Male	43	38.1
Female	70	62.0
Highest level of education (*n* = 114)		
Never attended	3	2.6
Primary	22	19.3
Secondary	78	68.4
University	3	2.6
Postgraduate qualification	2	1.8
Undisclosed	6	5.3
Employment status (*n* = 114)		
Employed full-time	21	18.4
Employed part-time	12	10.5
Unemployed or home duties	40	35.1
Student	0	0
Pensioner	37	32.5
Undisclosed	4	3.5
Usual method of transport to hospital (*n* = 111)		
Own transport	39	35.1
HealthNet bus	23	20.7
Minibus or taxi	15	13.5
Go George bus	13	11.7
Hired service	9	8.1
Walk	6	5.4
Driven by family member or friend	5	4.5
Ambulance	1	0.9
Clinic booked (*n* = 114)		
Ophthalmology	20	17.5
Orthopaedics	19	16.7
Surgery	12	10.5
Gynaecology	12	10.5
Oncology	10	8.8
Respiratory	7	6.1
ENT	6	5.3
Cardiology	6	5.3
Rheumatology	6	5.3
Family medicine	5	4.4
Metabolic	5	4.4
Urology	3	2.6
Colposcopy	2	1.8
Internal medicine	1	0.9
Estimated time taken to get from home to hospital (*n* = 107)		
< 15 min	14	13.1
15–29 min	16	15.0
30–59 min	22	20.6
60–119 min	32	29.9
120–179 min	13	12.2
≥ 180 min	10	9.4

*Source:* (CliniCom), Department of Health

† One person had the wrong participant code attached and therefore unable to determine gender.

There was a wide range of reasons given for missing OPD appointments (see Table 2). The most common reasons given were that participants were unaware that they had an appointment on that date (15.9%) or were confused about when the appointment was (10.6%), they were out of town at the time (10.6%), they were sick or admitted to hospital (9.7%) or they chose not to attend as they did not think it was needed (8.8%). Clerical issues both in OPD and in referring centres were raised, specifically, appointments being recorded for the participants incorrectly and participants being told appointments were cancelled but not being cancelled on CliniCom. Five participants contacted had actually attended their appointments, demonstrating an issue with attendance recording systems.

**TABLE 2:** Reasons for missing OPD appointments.

Reasons for missing appointment	Number	Percentage of participants (%)
Unaware of appointment date	18	15.9
Out of George	12	10.6
Confusion over appointment date	12	10.6
Sick or admitted to hospital	11	9.7
Chose not to attend	10	8.8
Family member sick or died	8	7.1
Clerical error – appointment should have been cancelled	8	7.1
Transport difficulties	7	6.2
Forgot about appointment	5	4.4
Patient attended appointment	5	4.4
Told by medical professional not to come	5	4.4
Attended but not seen	2	1.8
Another clashing appointment	2	1.8
Work commitments	2	1.8
Difficulty cancelling appointments	2	1.8
Miscellaneous†	4	3.5

*Source:* Personal communication with study participants

† Miscellaneous reasons were participant attended OPD, but queues were too long at admissions and OPD, so they left; too many appointments; participant felt unable to make it that day (no other reason given); and contradictory SMS messages meant participant thought appointment was cancelled.

When participants were asked whether they understood why they had an appointment at the hospital, 91.8% (*n* = 101) felt that they did, with 6.4% (*n* = 7) participants not understanding and 1.8% (*n* = 2) participants being unsure. When participants were asked whether they thought it was necessary for them to attend their appointment, 85.1% (*n* = 97) thought it was necessary, 9.7% (*n* = 11) thought it was not necessary and 1.8% (*n* = 2) were unsure.

Overall, 95.4% (*n* = 103) participants were satisfied with the OPD, 1.9% (*n* = 2) were unsure and 2.9% (*n* = 3) were not satisfied. Reasons given for being satisfied included the friendliness of staff, cleanliness and provision of high-quality medical care. The participants who were not satisfied gave the following reasons:

(1) advised by a doctor in OPD that they could be followed up at a local clinic rather than hospital
(2) unable to afford services
(3) felt the staff were rude regarding a psychiatric diagnosis.

Despite being satisfied overall with OPD services, issues such as waiting times and staff communication were raised multiple times.

Participants were asked what the hospital could do to make it easier to attend appointments. By far, the most common response was that the hospital should remind patients about appointments by SMS or phone call (*n* = 47, 44.8%). Thirty-nine percent (*n* = 41) of participants felt nothing could be improved. Other reasons included improved transportation (*n* = 8, 7.6%), reduced waiting times (*n* = 6, 5.7%), changes to how appointments are scheduled (*n* = 3, 2.9%), transfer of care to a more local clinic or hospital (*n* = 2, 1.9%) and easier cancellation systems (*n* = 2, 1.9%). Reasons given by one participant each included liaising with employers, liaising with family members and better communication with clinics. One participant also made a suggestion about appointment and transportation bookings that was already in place, suggesting a lack of awareness of the current systems.

## Ethical consideration

Ethics approval was obtained via Stellenbosch University Research Ethics Committee (N15/12/127).

## Discussion

The most common reason given for participants not attending was that they were unaware of their appointment. The majority of these participants had seen a clinician at a level-one hospital or clinic that had referred the participant, but the participant was not aware of being contacted by the hospital with the date. This was a feasible scenario given many of the referral systems requiring OPD ward clerks to contact patients with appointment information at a later date, often involving the referring centre to help contact the patient. A move towards giving patients an appointment date, when they are still sitting with the referrer would combat this. This was a new issue that had not been raised in previous research in South Africa or internationally. There were also related issues such as confusion over dates (10.6%) and appointments that should have been cancelled (7.1%), which could be improved with robust administrative systems.

The second most common reason was that the participant said they were out of the area at the time of appointment (10.6%), often in the Eastern Cape or the Karoo. This has been found to be a significant problem elsewhere.^7,8^ Although all participants said this was a temporary arrangement, multiple participants said it would be useful to have their care delivered closer to home. Given the wide geographical catchment area of GRH and the fact that almost 10% of participants would have travelled over 3 h to get to GRH, it is unsurprising that distance is a factor. More surprisingly is that issues that would be expected to play into this, such as transport, only came up infrequently (6.2%).

Although many of the findings of this study were in accordance with other research, factors that came up significantly less than other studies included forgetting appointments and transport issues.^8,9^ Financial difficulties came up in 95% interviews in one study^7^, yet were not given as a significant factor influencing missed appointments in any of these interviews.

SMS or phone reminder systems were a popular suggestion for improvement. The hospital currently uses SMS reminders in certain specialties, and this is something that could be rolled out through the OPD. However, strong evaluative frameworks are required when doing so, as evidence is inconclusive for SMS^13^ and phone^16^ reminders in South Africa. The fact that only 20% of participants who missed appointments were contactable on the given telephone numbers indicated this as a specific challenge to focus on. High cell phone turnover, reluctance to give accurate number for billing purposes and use of friends or family members’ numbers may all have contributed. This supports the recommendation of providing patients with appointment dates at point of care.

Based on the reasons given for missing appointments, approximately 50% of the appointments could have been cancelled, demonstrating a significant opportunity for improvement. The introduction of a toll-free telephone number or SMS system where patients were able to rebook or cancel appointments would encourage patients to inform the hospital of changes. This would allow them to rebook appointments, whereas many are currently getting lost in the system, and would allow other patients to use the appointment.

There were several limitations to this study. We were only able to contact 20% of eligible patients, potentially biasing the reasons given for non-attendance. To try and minimise this, we attempted to contact a larger sample, using various available contact numbers, and tried to phone people at different times of the day to reach the most representative group possible. The self-reporting nature of interviews is prone to social desirability bias. This may have created a bias towards hospital factors, rather than patient factors. However, similar studies have found that patients will admit to patient factors.^7,8,9^ Although semi-structured telephone interviews allowed us to reach a higher number of patients than other methodologies were likely to, it did not allow in-depth exploration of illness and health beliefs. These have been found to greatly influence attendance and would, therefore, be useful to explore further in the future.^12^

Some people have argued that staff and patient apathy may preclude meaningful DNA reductions.^11^ However, we found staff very motivated across specialties and external hospitals, and had multiple requests for specialty-specific analysis to target their main problems and the resources to replicate the study in other contexts. Although it is harder to assess patient motivation, the variable attendance across specialties suggests that motivation has a big impact. Many of the barriers, such as transport, are equally difficult for all patients but some clinics had a much higher non-attendance. Other studies showed that targeted interventions can have significant and persisting effects.^17^

Exploring reasons for non-attendance is difficult as few approaches are bias-free. In order to get as complete an understanding as possible of the challenges patients face in reaching their OPD appointments, it is important to address the question with multiple methodologies. Further quantitative analysis of some of the demographic and clinical factors would be beneficial, including outcomes such as distances travelled, age, gender and previous number of appointments attended. If specific at-risk groups were targeted through this, then more in-depth interviews and focus groups could be used to get a more thorough understanding of their challenges. This could include more of a focus on health and illness beliefs, which was difficult to obtain in this study.

## Conclusion

The aim of this study was to explore factors contributing to patients missing their OPD appointments and patients’ ideas of how to improve the accessibility of OPD, allowing the development of a more patient-centred health service. A greater understanding of reasons behind non-attendance has allowed more targeted interventions to maximise attendance, addressing both patient and hospital factors.
